# The effect of problem-based learning in patient education after an event of CORONARY heart disease – a randomised study in PRIMARY health care: design and methodology of the COR-PRIM study

**DOI:** 10.1186/1471-2296-13-110

**Published:** 2012-11-20

**Authors:** Anita Kärner, Staffan Nilsson, Tiny Jaarsma, Agneta Andersson, Ann-Britt Wiréhn, Peter Wodlin, Lisa Hjelmfors, Pia Tingström

**Affiliations:** 1 Department of Social and Welfare studies (HAV), Linköping University, Linköping, Sweden; 2 Vikbolandet Health Care Center, Primary Health Care in eastern Östergötland, County Council of Östergötland, Norrköping, Sweden; 3 Local Health Care Research and Development Unit, County Council in Östergötland, Linköping University, Linköping, Sweden; 4 Department of Cardiology, County Council of Östergötland, Linköping University Hospital, Linköping, Sweden; 5 Faculty of Health Sciences, Linkoping University, Linköping, Sweden; 6 Department of Medical and Health Sciences (IMH), Linkoping University, Linköping, Sweden

## Abstract

**Background:**

Even though there is convincing evidence that self-care, such as regular exercise and/or stopping smoking, alters the outcomes after an event of coronary heart disease (CHD), risk factors remain. Outcomes can improve if core components of secondary prevention programmes are structurally and pedagogically applied using adult learning principles e.g. problem-based learning (PBL). Until now, most education programs for patients with CHD have not been based on such principles. The basic aim is to discover whether PBL provided in primary health care (PHC) has long-term effects on empowerment and self-care after an event of CHD.

**Methods/Design:**

A randomised controlled study is planned for patients with CHD. The primary outcome is empowerment to reach self-care goals. Data collection will be performed at baseline at hospital and after one, three and five years in PHC using quantitative and qualitative methodologies involving questionnaires, medical assessments, interviews, diaries and observations. Randomisation of 165 patients will take place when they are stable in their cardiac condition and have optimised cardiac medication that has not substantially changed during the last month. All patients will receive conventional care from their general practitioner and other care providers. The intervention consists of a patient education program in PHC by trained district nurses (tutors) who will apply PBL to groups of 6–9 patients meeting on 13 occasions for two hours over one year. Patients in the control group will not attend a PBL group but will receive home-sent patient information on 11 occasions during the year.

**Discussion:**

We expect that the 1-year PBL-patient education will improve patients’ beliefs, self-efficacy and empowerment to achieve self-care goals significantly more than one year of standardised home-sent patient information. The assumption is that PBL will reduce cardiovascular events in the long-term and will also be cost-effective compared to controls. Further, the knowledge obtained from this study may contribute to improving patients’ ability to handle self-care, and furthermore, may reduce the number of patients having subsequent CHD events in Sweden.

**Trial registration:**

NCT01462799

## Background

Despite impressive progress in treatments, coronary heart disease (CHD) is still a major cause of death among men and women in most European countries [1]. In Sweden 41 000 individuals suffer a myocardial infarction (MI) every year [2]. About 60% who survive an MI or related coronary event have a high risk of having another cardiac event [3]. However, these outcomes can be improved by lifestyle changes, e.g. smoking cessation and/or starting regular exercise, as suggested in European guidelines, [4]. Further, medication using e.g. beta –blockers can lead to a 19-48% decrease in mortality and a 28% decrease in reinfarction rates [5]. Interestingly, the INTERHEART study [6] showed that nine modifiable factors explain a large (> 90%) proportion of the threat of developing an initial acute MI. These are smoking, elevated ApoB/ApoA1 ratio, history of hypertension, diabetes, abdominal obesity, psychosocial factors, daily consumption of fruits and vegetables, regular alcohol consumption and regular physical activity [7]. European guidelines emphasise: avoidance of smoking and overweight, physical exercise (at least 30 min/day), healthy food, blood pressure < 140/90 mm Hg, total cholesterol < 5 mmol/l [1]. Smoking cessation, intake of fruit and vegetables and exercise can together lower the relative risk of MI by up to 80% [7]. Although this is known, of more than 4300 asymptomatic patients with CHD, 17% continue to smoke; 43% are obese; 70% have elevated blood pressure, and 66% have elevated total serum cholesterol about six months after starting medication for hypertension and/or high blood lipids, as analysed retrospectively [8]. To address the effectiveness of multifactorial lifestyle interventions, a study systematically reviewed 25 randomised controlled trials (~70700 patients) in primary and secondary prevention of CHD and type 2 diabetes [9]. The evidence for the interventions was weak overall. The trials were few, samples were small, and the intensity of the interventions was surprisingly low according to the authors predefined minimum level of 60-min intervention as an inclusion criterion. Thirteen of the 25 studies used low- (11-30 h total) or very low intensity interventions (1-10 h total). The cholesterol levels and blood pressure did not differ between the groups (controls received the usual care). However, body mass index (BMI) was positively affected, as indicated by significantly lower results in lifestyle intervention groups compared to controls. Although the evidence was limited, significant improvements in self-reported risk behaviour were identified regarding at least two of the three key aspects of healthy diet, namely physical activity and stress management. The interventions were considered to have a relevant effect on clinical outcomes such as mortality, cardiac events or hospitalisation. The authors point out the need for further trials to describe interventions transparently regarding, for example, concepts, duration, delivery and adherence. Three levels of information should be included: 1) patient attendance and participation, 2) behavioural change regarding diet, physical exercise and stress management, and 3) clinical/laboratory outcomes. Another recent systematic review [10] of more than 10,000 patients with CHD showed improvements by lifestyle interventions in dietary and exercise outcomes but no overall effect on smoking. However, the authors state that the poor quality of the trials made it difficult to come to concrete conclusions, thus further research is emphasised.

In Sweden, after a CHD event, patients are offered brief cardiac rehabilitation in hospital care, and stable patients are thereafter referred to primary health care (PHC). Self-care goals are, however, not identified or followed-up structurally in PHC, and therefore cannot accurately be supported. Patients’ beliefs about CHD and its medication vary qualitatively and may not lead to healthy choices as patients sometimes consider CHD as impossible to affect. For example, they may have fatalistic views, may describe smoking as health promoting, or seldom mention medication as a way of improving the prognosis [11]. Another aspect that affects the accomplishment of self-care goals is linked with hindering or facilitating factors in life [12]. According to Bandura’s social cognitive theory of self-regulation, beliefs in one’s own capabilities to organize and execute the courses of action required to handle situations in future influence how people think, feel motivated and act [13]. Learning that is based on patients’ beliefs is necessary for effective patient education [14,15]. Until now, most education programs for patients with cardiac problems have not included the patients’ beliefs and have not been based on adult learning principles including that adults need to know what, how and why they learn. They need to have attention; identify earlier knowledge, and feel motivated to learn. Most pedagogical processes in health care seem to be unplanned and embedded in treatment; the goals are vague or non-existent [16]. However, self-care may improve if structured pedagogical education based on adult learning principles [17] e.g. problem-based learning (PBL), is applied. The basic ideas of PBL are to: have an investigative approach to learning, take responsibility for the learning, use real-life situations, reflect on one’s own learning [18,19]. PBL can empower patients to improve their self-care [20] by helping them become more active in self-management of their illness, and choosing to change their behaviour [21]. Group education also seems to be of importance. Two Scandinavian systematic reviews of patient education in diabetes management [22], type 2-diabetes and chronic obstructive pulmonary disease (COPD) [23] found that group education led by people with expertise, skilled in the chosen educational model, improved knowledge, empowerment or self-efficacy to manage the disease. The group education led to clinically important improvement of long-term glycated haemoglobin (HbA_1c_) compared to individual education. In COPD a higher degree of self-management in severe situations due to the disease was found and the education also contributed to fewer COPD-related deaths. According to a Swedish study, joining peer-support groups after CHD resulted in more regular exercise, less smoking, a closer network and more social support compared to those who declined participation in such groups [24]. However, the literature shows inconsistency regarding the benefits of group-based support, which not did change HbA_1c_, cholesterol, blood pressure and well-being at two-year follow-up. The peer supporters showed a decline in well-being at follow-up, implying that this role could be demanding and stressful [25]. This may suggest that peer support is relevant and that group education should be provided by health care personnel skilled in the educational model chosen for the project. Another publication showed that 20 sessions of cognitive behavioural therapy , led by nurses or educational therapists in PHC and oriented towards educating and motivating the patients, reduced fatal and non-fatal CHD by 41%, and also improved future optimism [26]. Tingström’s study using PBL as an educational base after CHD also significantly improved hope for the future and improved patients’ knowledge about their illness more, compared to controls, after one year of education [27].

It is a challenge to identify what kind of program is most effective and to what level of intensity it should be provided. The literature and the knowledge of the long-term effects of PBL on self-care after CHD have not been scrutinised or rigorously evaluated, and this constituted the rationale for designing a randomised study. Our basic aim is to discover whether PBL provided in *primary* health care has long-term effects on empowerment and self-care after an event of *coronary* heart disease (COR-PRIM). In COR-PRIM, PBL as a foundation for the learning process will be tested against a control group that will be informed by predetermined, written patient information in a structured way based on the traditional model of information transferred to individuals. This will be accomplished to evaluate what type of education is required to affect patient empowerment [21], self-efficacy and beliefs in lifestyle changes [13]. An underlying principle of this project is to identify patients’ beliefs about self-care, and to incorporate these as an effective starting point for PBL patient education in PHC.

### Study hypothesis

The hypothesis of the COR-PRIM study is that one year of PBL in patient education improves a patient’s beliefs, self-efficacy and empowerment to change self-care significantly more compared to one year of standardised home-sent patient information.

## Methods/Design

### Study design

**Figure 1:**
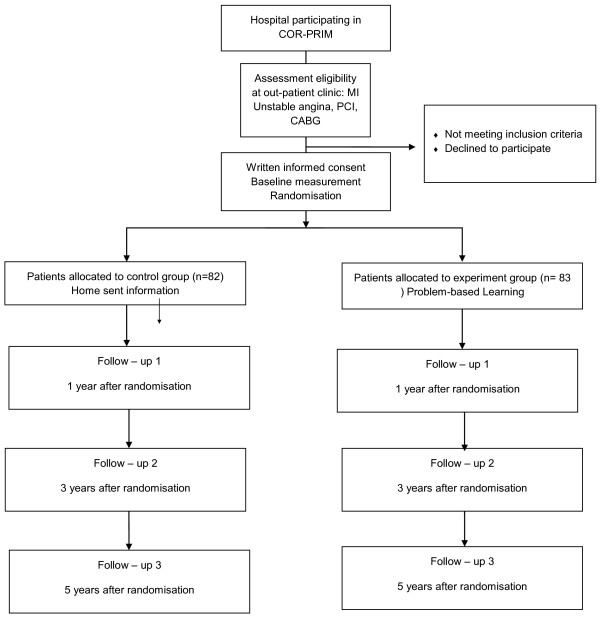
Study design.

A randomised controlled design will be used in this parallel-group study (*see* Figure 1) including 165 patients with CHD. Half of the patients will be randomised to an experimental group (PBL) and half to a control group (home-sent patient information). The study complies with the Declaration of Helsinki and was approved by The Regional Ethical Review Board in Linköping, Dnr 2010/128-31.

#### Nurses training

The selection and training of nurses is fundamental to this project. The goal is to recruit district nurses working in PHC and if possible with experience of patients with CHD. The nurses will be offered the opportunity to take part in a training session for two days given by the project team. The training involves learning about tutoring, with a focus on the central characteristics of PBL e.g. learner-centred, self-directed, real life situations and problem-solving [28]. Starting points that will be used to trigger the patient’s problem-solving process will be developed by the nurses in collaboration with the research team. Later on, the nurses will be tutored monthly by the first and last author during the whole process in order to discuss and develop their work. The nurses will also take part in seminars reporting on findings e.g. patients’ beliefs about self-care and the enactment of PBL in the groups, throughout the study.

### Study population

#### Patients and study site

Eligible patients will be identified at the heart unit, Vrinnevi hospital in Norrkoping, Sweden, from the electronic medical record based on the following criteria. The inclusion criteria in the study are: patients of all ages with CHD verified by MI and/or Percutaneous Coronary Intervention (PCI) and/or coronary artery bypass surgery (CABG) within 12 months before the planned start of the intervention. Patients should be stable regarding their cardiac conditions and have optimised cardiac medication that has not substantially changed during the last month; they should have completed heart school in hospital care (if applicable); and should be listed at one of six specific PHC centres that have agreed to join the project at the time of inclusion. Exclusion criteria from the study are: planned CABG or other conditions demanding continued cardiologist care; e.g. on-going contact with heart failure clinic due to drug titration or investigations, e.g. myocardial scintigraphy to detect ischemia before a new PCI; life expectancy ≤ one year; documented psychiatric disease causing difficulties cooperating with other people; or obvious abuse of alcohol or narcotics. Patients will also be excluded if they are unable to communicate or read the Swedish language and if they are participating in other studies affecting the results.

### Conventional care and interventions

#### Conventional care

After the hospital care, all patients will be offered individual information about CHD, self-care and treatment. Cardiac rehabilitation will also be accessible at an outpatient clinic at the hospital. This will involve counselling visits with a nurse and a cardiologist about four weeks and 6–12 months after discharge respectively; physical exercise 1–2 times per week, for 3–4 months; and diet counselling. In addition, there is also a heart school for one day, mainly focussing on CHD, physical exercise, stress, diet and medication. If the condition permits, i.e. the patient’s symptoms are stable, patients will be referred to PHC. Follow-up will be provided by a general practitioner (GP) if needed due to issues associated with blood pressure, blood lipids and smoking. Thereafter, follow-up by the GP will be offered yearly in most cases.

#### Intervention group PBL

These patients will receive PBL patient education in PHC to support self-care (see below), and also conventional care as described above.

The overall goal of the education is to improve self-care through strengthened empowerment

Partial goals are to:

Understand the health benefits of lifestyle changes that the patients want to accomplish

Cope with situations challenging accomplishment of lifestyle changes

Understand and cope with:

Symptoms (angina pectoris, dyspnoea, swelling legs, tiredness)

Changes in diet, physical exercise, medication, psychosocial factors, sexual life

Mental reactions of CHD (depression, anxiety, fear)

Working life (vocational training, stress, dynamic and static work)

Patient education according to the principles of PBL will be provided to a group of 6–9 patients, meeting for a total of 13 occasions for two hours. This will be every week for the first month, then for the next two months there will be two meetings/month and at 16, 20, 26, 39 and 52 weeks after the start. The PBL-intervention will be completed one year after the start.

**Figure 2:**
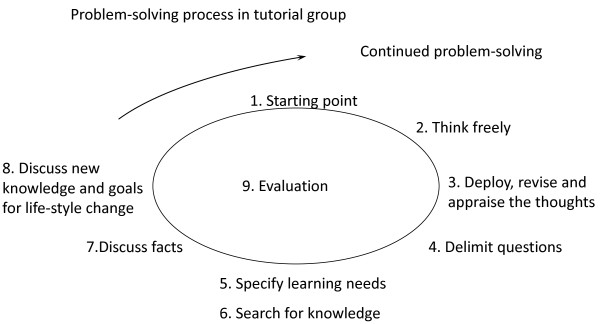
PBL process.

A PBL model (Figure 2) [27] supporting the patients learning about self-care will be used. A study guide for the patients will be provided in which learning- and self-care goals may be documented by the patients.

At each meeting the patients use triggers e.g. pictures, texts or concrete materials as starting points for their learning process

District nurses work as tutors after completion of a tutorial education. They support the patients in formulating issues, shared learning goals- and individual self-care goals.

Resource persons, e.g. a physician, physiotherapist, dietician, or social worker, could be invited to respond to questions not solved by the patients themselves. Relatives/family members could also be invited to these meetings.

During the last meeting, focus-group interviews will be performed to collect data about patients’ beliefs, their performance of self-care, and their experiences during the study.

#### Control group receiving home-sent patient information

These patients will serve as controls. They will receive conventional care as well as the following patient education to support self-care.

Patient education about self-care according to predetermined written patient information [29] will be provided to a group of patients consisting of 6–9 people at a meeting in PHC directly following randomisation and again after one year.

Events linked to the study during the year will be presented at the first meeting, for example regarding the background and aims of the study, the distribution of patient information, and follow-up measurements.

Written patient information will be mailed to the patients’ homes at the same times as the PBL meetings.

During the last meeting, focus-group interviews will be performed to collect data about the patients’ beliefs, their performance of self-care, and their experiences during the study.

Material used in both groups will include a patient diary to document experiences of self-care, and brochures produced e.g. by The Swedish Heart and Lung Foundation.

### Study outcomes and assessment

To determine whether the pedagogical methodology, called PBL, is practicable for achieving self-care goals in the long-term compared to standardised home-sent patient information, study outcomes on achieving self-care goals of patients with CHD will be performed by analysing and comparing patients’ beliefs, self-efficacy, and empowerment to make changes in self-care, considering patients using PBL and those using home-sent patient information. Also, to determine the effects of PBL on reducing new cardiovascular events, smoking, blood pressure, BMI, waist measurement, HbA1C, fp-Glucose, plasma lipids, cost-utility and health care consumption by comparing the outcome in PBL-groups with the outcome in control groups (home-sent patient information). Following verification of appropriateness and informed consent, patients’ baseline characteristics will be collected from the Swedeheart® register and medical chart, interviews, observations and questionnaires (*see* Table 1). The questionnaire SWE-CES-10 was developed to survey empowerment in patients with CHD. This questionnaire was originally based on SWE-DES-23, which is a valid and reliable tool to assess empowerment in diabetes and rheumatic disease [30,31]. SWE-DES-23 was tested and shortened to become SWE-DES-SF-10 and found to be valid and reliable in relation to the original version. The items were general in their nature and not disease-specific, and this allowed us to contact the creator who authorized an adaptation by replacing the word ‘diabetes’ with ‘heart disease’ in all 10 items. The SWE-CES-10 is a self-administered questionnaire. Follow-up assessments will take place one, three and five years after randomisation. Data will be collected in PHC and interviews will be performed at locations chosen by the patients.

**Table 1:** Measurement scheme

**Variable**	**Instrument**	**Baseline**	**1 year**	**3 years**	**5 years**
Primary outcome					
a. Empowerment	Swe-CES-10	X	X	X	X
Secondary outcomes					
b. Self-efficacy	GSES, NSES,	X	X	X	X
c. Physical exercise	PSES , IPAC , Stages of change scale	X	X	X	X
d. Well-being	Ladder of life, EQ5D	X	X	X	X
e. Risk factors	Fp-Cholesterol, fp-HDL, fp-LDL, fp-Triglycerides, HbA1c, fp-Glucose, blood pressure, smoking, BMI, waist measurement	X	X	X	X
f. Experiences of self-care	Reflective diary		X		
g. Beliefs about self-care	Focus group interviews	X	X		
h. Enactment of PBL	Observations, interviews and documents		X		
Feasibility of the intervention			X		

This table provides an overview of the time schedule of all measurements, and ways to collect data.Questionnaires: Swe-CES-10: The Swedish version of the coronary empowerment scale is a modified version of the Swedish version of the diabetes empowerment scale-23 [31]; GSES: The General Self-Efficacy Scale [32], NSES: Nutrition Self-Efficacy Scale [33], PSES: Physical Self-Efficacy Scale [33], IPAC: International Physical Activity Questionnaire [34]; Stages of change Scale [35,36]; Ladder of life [37,38];Swedish version of EQ5D: EuroQol [43].Qualitative analysis: Experiences of self-care documented in reflective diary and analysed by critical discourse analysis [40]; Beliefs of self-care collected in focus groups [48] and analysed by qualitative content analysis [39]; enactment of PBL collected by different methods and using ethnographic analysis [41,42].

Primary and secondary outcome measurements will be assessed at baseline and, at one, three and five years after randomisation.

#### Primary outcomes

The primary outcome is empowerment to reach self-care goals one year after randomisation.

#### Secondary outcomes

The secondary outcomes to be measured in this study in order to determine the long-term effect of PBL versus home-sent patient information regarding self-care are: self-efficacy in general [32], healthy diet [33] and physical exercise [34-36]; well-being [37,38]. Changes in patients’ beliefs about self-care will be assessed using qualitative content analysis [39]. Patients’ experiences of self-care documented in a reflective diary by the patients will be qualitatively analysed by critical discourse analysis [40]. The enactment of PBL as an educational model will be assessed by interviews, participant observations and field notes using ethnographic analysis [41,42]. New cardiovascular events, blood pressure, BMI, waist measurement and blood tests will be followed-up to objectively measure effects of self-care. Also, costs will be calculated from a health care perspective. Data on costs will be collected prospectively throughout the study for the PBL strategy as well as for the home-sent information strategy. Cost data will include, for example, personnel costs for staff conducting the programmes for PBL-intervention and the home-sent information as well as material costs for each approach (fixed and variable costs). Each activity will be measured in minutes and thereafter priced using relevant unit costs for each item.

For the cost-utility analysis the effect data are quality-adjusted life years (QALYs). The EQ-5D [43] will be used to measure health-related quality of life. We will also use effect data in a cost-effectiveness analysis, using the SWE-CES-10 score as an effect measurement after one year.

Health care consumption will be compared for the PBL-intervention group and the home-sent information group receiving home-sent patient information using data collected by the county council.

### Sample size

The sample size was determined for testing whether the difference in mean values between the randomised groups differed from 0 concerning the values in the empowerment instrument, SWE-DES – 23 scale [31]. The mean value in the group randomised to having home-sent patient information was expected to be 3.0 (standard deviation = 1.2), whilst the analogue value for those randomised to receiving PBL-education, was 3.6 (standard deviation = 1.2). At a significance level of 5% and a power of 80% this yielded the required sample size in each group of at least 63. The clinical significance of 0.6 in the main outcome score is based on the estimation of discriminant validity showing that patients with diabetes, who could be compared with patients with CHD in terms of life-long disease, who reported poor self-rated health scored around 3 in several subscales of SWE-DES – 23 scale; corresponding figures for those who reported good self-rated health was around 0.6 above 3 [31]. Losses to follow up and missing values will, if reasonably random or not too extensive, be taken care of by substituting mean or median values of real data that are typical for the sample [44]. We will allow for 10% attrition due to losses to follow-up, and death.

### Study organisation and randomisation

Nurses at the outpatient clinic at Vrinnevi hospital involved in cardiac rehabilitation will identify eligible patients. The patients will receive information about the study by mail, and then, after about two weeks, will be contacted by a researcher to get personal information about what enrolment in the study could mean. The patients will be given the opportunity to ask the researcher questions about the study.

Patients will be enrolled in the study following baseline assessment and written informed consent. In order to initiate one experiment and one control group, 12–18 patients are needed. Randomisation will be carried out, with sealed unmarked opaque envelopes, which will be assigned to an administrator in a room separate from the research and intervention area. By using a block of 18 study numbers, that will be blindly allocated to either the experiment group (PBL–intervention) or the control group (home-sent patient information) [45]. The envelopes will contain a card with a unique number from 1 to 185. The administrator will be blinded during the randomisation process. The outcome assessors will be blinded during analysis of new cardiovascular events. The patients and nurses/tutors will not be blinded as the patient education as the PBL education obviously differs from the home-sent information.

### Analysis

All analyses will be conducted according to the intention-to-treat- principle. Appropriate quantitative methods for parametric analyses regarding normally distributed, continuous data will be compared by using e.g. Student’s *t* test and non-parametric analysis for data that is not normally distributed will be performed. The primary variable ‘empowerment to reach self-care goals’ will be evaluated using non - parametric methods, e.g. a Mann–Whitney U test. *P*-values below 0.05 will be considered as statistically significant.

The project started in November 2010 with a pilot study to identify the feasibility of the intervention [46]. The result of the pilot study was that it was feasible to organize the study in PHC. Fifty-three per cent (n = 17) of eligible patients joined and were randomly allocated according to the design. The pilot study involves a small- scale ethnographic element [47] with the aim of identifying and describing the enactment of PBL-processes in the experiment group using PBL. The collection of all data in the pilot study (one-year follow-up) was completed in December 2011.

The main study started in September 2011 and the first study patients were included. We are planning to enrol patients during 2011-2014 and follow-up will continue until 2019. The first finding of the COR-PRIM study will become available in 2014, and the first results of the main study around 2015.

## Discussion and conclusion

Secondary prevention may positively influence risk factors and thus also reduce the number of recurrent coronary events. In the long term, secondary prevention requires co-operation between hospital care and PHC. Not considering the gap between these caring levels impairs the quality of care. Current strategies for secondary prevention do not work optimally since adult learning principles are not used and patients are not involved in their own goal setting. In the proposed study we will test the effect of a group-based adult learning method - that of PBL. This method is expected to reduce the number of secondary cardiovascular events in the long-term and also be cost-effective compared to home-sent patient information. Long-term follow-up is important for finding out how such interventions affect the patients’ future health.

## Competing interests

The authors declare that they have no conflicting interests.

## Authors’ contributions

AK, TJ, SN and PT designed the study. AA designed the health economics part of the study. A-BW is responsible for statistical methods. AK drafted the manuscript and all authors contributed to the final concept. PW has made a substantial contribution to formulating inclusion- and exclusion criteria and assessments. LH contributed the ethnographic part of the pilot study. All authors have read and approved the final manuscript.

## Pre-publication history

The pre-publication history for this paper can be accessed here:

http://www.biomedcentral.com/1471-2296/13/110/prepub
